# Detection of pulmonary ground-glass opacity based on deep learning computer artificial intelligence

**DOI:** 10.1186/s12938-019-0627-4

**Published:** 2019-01-22

**Authors:** Wenjing Ye, Wen Gu, Xuejun Guo, Ping Yi, Yishuang Meng, Fengfeng Han, Lingwei Yu, Yi Chen, Guorui Zhang, Xueting Wang

**Affiliations:** 1 Department of Respiratory Medicine, Xinhua Hospital, School of Medicine, Shanghai Jiao Tong University, Shanghai, 200092 China; 2 School of Electronic Information and Electrical Engineering, Shanghai Jiao Tong University, Shanghai, 200240 China; 3 Department of Radiology, Xinhua Hospital, School of Medicine, Shanghai Jiao Tong University, Shanghai, 200092 China; 4 Shanghai Jiaotong University School of Medicine, Shanghai, 200092 China

**Keywords:** Pulmonary nodule, Ground-glass opacity, Deep learning, Artificial intelligence, Computer-aided diagnosis

## Abstract

**Background:**

A deep learning computer artificial intelligence system is helpful for early identification of ground glass opacities (GGOs).

**Methods:**

Images from the Lung Image Database Consortium and Image Database Resource Initiative (LIDC-IDRI) database were used in AlexNet and GoogLeNet to detect pulmonary nodules, and 221 GGO images provided by Xinhua Hospital were used in ResNet50 for detecting GGOs. We used computed tomography image radial reorganization to create the input image of the three-dimensional features, and used the extracted features for deep learning, network training, testing, and analysis.

**Results:**

In the final evaluation results, we found that the accuracy of identification of lung nodule could reach 88.0%, with an F-score of 0.891. In terms of performance and accuracy, our method was better than the existing solutions. The GGO nodule classification achieved the best F-score of 0.87805. We propose a preprocessing method of red, green, and blue (RGB) superposition in the region of interest to effectively increase the differentiation between nodules and normal tissues, and that is the innovation of our research.

**Conclusions:**

The method of deep learning proposed in this study is more sensitive than other systems in recent years, and the average false positive is lower than that of others.

## Background

Pulmonary ground-glass opacity (GGO) is defined as a hazy opacity that does not obscure the underlying bronchial structures or pulmonary vessels on high-resolution computed tomography [1]. GGOs can be observed in benign conditions, such as focal interstitial fibrosis, inflammation, and hemorrhage; preinvasive lesions, such as atypical adenomatous hyperplasia and adenocarcinoma in situ; and malignancies, such as minimally invasive adenocarcinoma and lepidic-predominant invasive adenocarcinomas [2]. Lung adenocarcinoma is the most common histologic subtype of lung cancer and shows high heterogeneity at the histological and cellular levels [3]. Patients with lung adenocarcinoma are usually diagnosed when they are in the advanced stages, and median survival time after diagnosis is usually less than 1 year [4]. The extent of a malignant GGO correlates with the prognosis after surgical resection. The computed tomography (CT) value of GGOs is always lower than that of blood vessels; therefore, GGOs may not always be obvious on CT images, and they may be missed. The recognition of GGO is based on a subjective assessment of lung attenuation at CT, but observation of pulmonary nodules by doctors is labor-intensive and time-consuming, and because of personal differences, the results of examination may often be different.

President Obama proposed the “precision medicine plan” in his State of the Union address in 2015 [5]. Precision medicine is a new medical concept and the model is based on individualized medical treatment; this concept has been spurred by the rapid progress of genome sequencing technology and the cross application of biological information and large data science. This is a new emerging field of medicine. With the advent of “big data”, more accurate diagnosis and identification of lung nodules, especially GGOs, is possible. It is now feasible to use medical data for diagnosis and treatment of lung cancer; this could greatly improve the survival rate of lung cancer patients.

The principle of computer-aided detection (CAD) includes applying a mathematical model and data programming to medical diagnosis. It has the ability to quickly (in near real-time) perform analytical computations on digital information; moreover, the errors with manual operation due to fatigue and individual judgment differences are avoided. Through imaging, computer analysis, and calculation, CAD has various applications such as diagnosis of breast lesions, CT virtual colonoscopy, diagnosis of liver disease, and diagnosis of brain tumors on magnetic resonance imaging. In recent years, there has been some progress in CAD technology with regard to detecting lung nodules on CT images [6]. Deep reinforcement learning combines the perceptive ability of deep learning with the decision-making ability of intensive learning. It is a form of artificial intelligence that is closer to the human thought pattern. At present, deep learning is being used for lesion classification, segmentation, and recognition [7, 8].

Artificial intelligence is an important branch of computer science. It is regarded as one of the three leading technologies in the world. The main research fields of artificial intelligence include machine perception, machine thinking, and machine learning and behavior. Deep learning is an important emerging field of artificial intelligence in recent years that has seen much new advancement in recent times. Deep learning allows computational models that are composed of multiple processing layers to learn representations of data with multiple levels of abstraction. It discovers intricate structure in large data sets by using the back propagation algorithm to indicate how a machine should change its internal parameters that are used to compute the representation in each layer from the representation in the previous layer. Deep learning can extract features from training images to improve the accuracy of prediction [9–11].

Taking into account the current global research status, we propose to establish an artificial intelligence system for the evaluation of pulmonary nodules, especially GGOs, which are difficult to diagnose. Early identification of GGOs will have higher diagnostic value and would be beneficial for the early detection of lung cancer.

## Methods

### Data sources

We used the Lung Image Database Consortium and Image Database Resource Initiative (LIDC-IDRI) database as the source of pulmonary nodule data. This database has the largest number of public lung images, and contains complete lung CT image slices and the specific annotation information of all nodules in image slices from 1007 patients. The LIDC-IDRI database was collected and published by the American National Cancer Institute to serve as an international research resource to aid research of early lung cancer [12]. Each patient has an eXtensible Markup Language (XML) format file. These files contain detailed information regarding the number of pulmonary nodules, their location, and characteristics, as interpreted by four radiologists. The characteristics deemed appropriate for diagnosis of pulmonary nodules include subtlety, internal structure, calcification, sphericity, margin, lobulation, spiculation, texture, and malignancy [13]. LIDC–IDRI includes all types of pulmonary nodules, such as solid nodules, part-solid nodules, and ground glass nodules. This database was used by the computer for the deep learning process to identify nodules.

For our study, all the GGO images in the database were extracted based on the characteristics of internal structure and texture [13]. Besides, Xinhua Hospital also provided 221 GGO images of 154 patients from 2016 to 2017 to expand the sample size. The cases of GGOs provided by Xinhua Hospital were identified and confirmed by two radiologists and two respiratory physicians, to ensure accuracy.

### Pulmonary region extraction

**Fig. 1:**
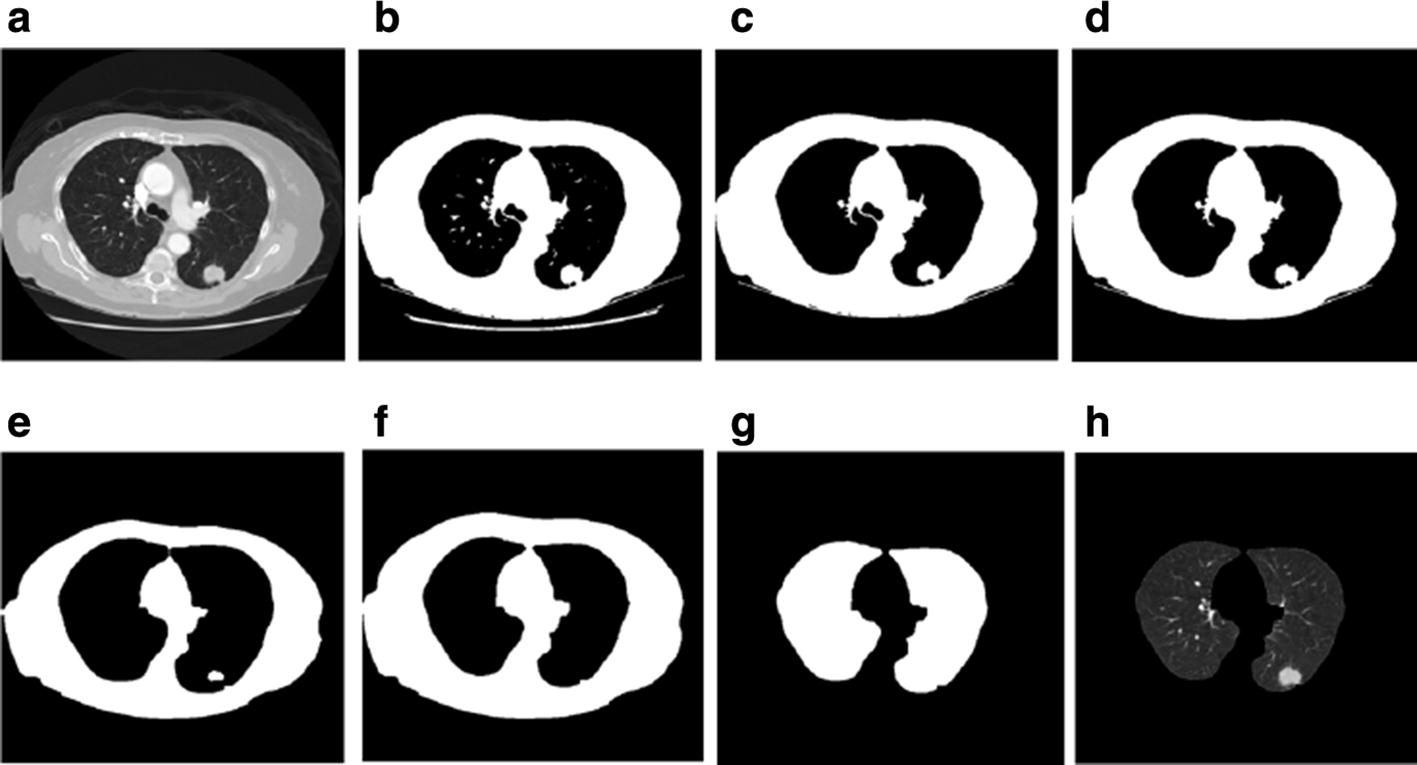
Steps of pulmonary parenchyma extraction. **a** Original CT image; **b** binarization of the CT image; **c** preliminary extraction of pulmonary contour; **d** filling airways; **e** contours corrosion; **f** contour mending and expansion; **g** pulmonary masking; **h** pulmonary parenchyma image

On the CT image, the pulmonary parenchyma includes the bronchus and bronchoalveolar structures. We analyzed CT slices to identify pulmonary nodules; therefore, we only focused on the lung parenchyma, and not on the external contour. In order to minimize the error of the external contour on the experimental results, the lung parenchyma was extracted by threshold binarization, extraction of the maximum connected component, and separation of the adhesions between the pulmonary nodules and pleura and the pulmonary contour by means of corrosion (Fig. 1). Only the lung parenchyma was retained for subsequent analysis.

### Nodule extraction

**Fig. 2:**
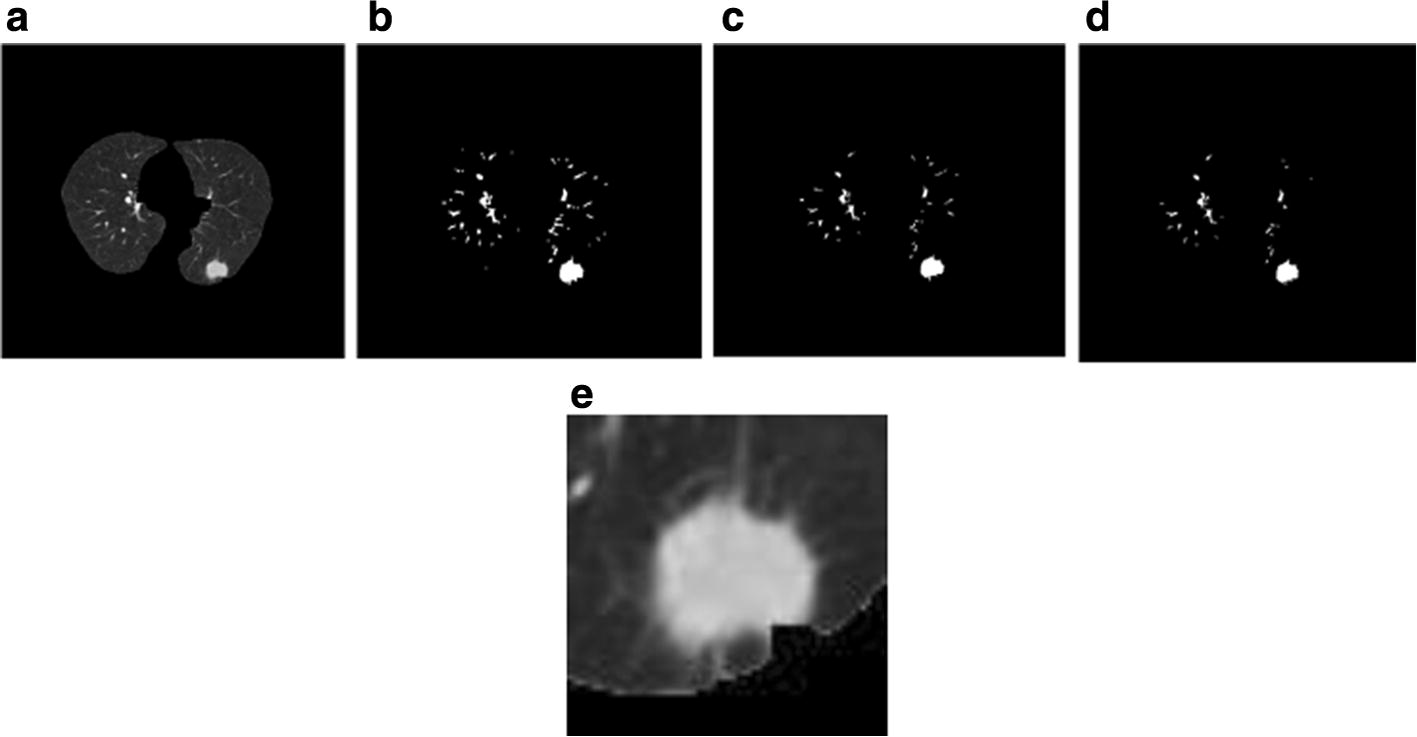
Steps of region of interest (ROI) extraction. **a** Pulmonary parenchyma image; **b** lung parenchyma after threshold processing; **c** deletion of a small structure; **d** deletion of a thin long structure; **e** region of interest image

After analysis and extraction of the pulmonary parenchyma, we could determine the position of the candidate nodule. Taking the centroid location as the center, we cut out 64 * 64 small blocks from the lung parenchyma, which were regarded as the regions of interest (ROIs) (Fig. 2).

### ROI superposition

**Fig. 3:**
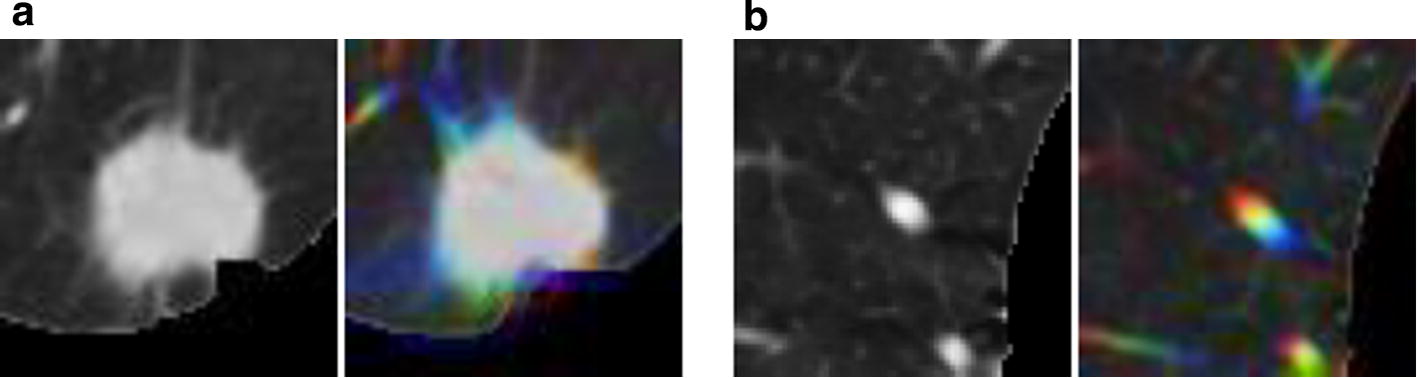
Region of interest (ROI) superposition. **a** ROI and red, green, blue (RGB) superposition effect of pulmonary nodules. **b** ROI and RGB superposition effect of normal lung tissue

Large nodules were easy to find; however, some smaller nodules and GGOs were similar to normal lung tissue on the image. In our study, in order to better differentiate between the pulmonary nodules (especially smaller nodules) and normal lung tissue, we used three continuous CT slices to attain more features of the ROI. After the centroid of a candidate pulmonary nodule was obtained, upper and lower CT slices were extracted from the initial CT slice, the three ROI pieces were superimposed on red, green, and blue channels (RGB), respectively, and pseudo color images were formed. Because of the spheroidal characteristics of pulmonary nodules, the three consecutive slices could be approximately overlapped, and the superposed RGB images were also spherical. Normal tissues, such as blood vessels, were seen as longitudinal stripes, and most of them were not perpendicular to the horizontal surface; therefore, they had a distinct RGB change after superposition (Fig. 3). After RGB channel superposition of the ROI, we could see the longitudinal trend of some tissues on 2D image. This method significantly enhanced the differentiation between pulmonary nodules and normal tissue.

### Deep learning

In this study, we used AlexNet [14] and GoogLeNet [15] to detect pulmonary nodules, and ResNet50 [16] to detect GGOs. Convolutional architecture for fast feature embedding (CAFFE) [17] was used as the framework for deep learning; it was developed by Berkeley Vision and Learning Center. CAFFE has the advantages of fast operation and high extensibility. The operating system was CentOS 7.3 and the GPU video card used was GeForce GTX 1080 N (NVIDIA, Santa Clara, CA).

### Deep learning of pulmonary nodules

We used the LIDC–IDRI database as a sample of CT slices for deep learning training. We used more than 10,000 ROI pseudo color images of pulmonary nodules extracted from the CT slices of 800 patients and about 12,000 ROI pseudo color pieces of normal pulmonary tissue as a training sample set. After we achieved a prediction model through deep learning training, another 176 patients’ CT images from the training sample set were used as the testing sample set. There were 321 pulmonary nodules in the testing sample set.

### Deep learning of GGOs

We extracted 1293 ROI pictures of GGOs from the LIDC–IDRI database based on nodule characteristics in the XML files and confirmation by two radiologists and two respiratory physicians. Of the 1293 samples of GGOs, 1000 ROI pictures were placed in the training set and 293 in the testing set. Because of the small sample size of GGOs in the LIDC–IDRI, Xinhua Hospital also provided another 221 pictures of GGOs from 154 patients to expand the training set. Finally, there were 1221 pictures of GGOs and 1200 of non-GGOs in the training set, and 293 pictures of GGOs and 300 of non-GGOs in the testing set.

**Fig. 4:**
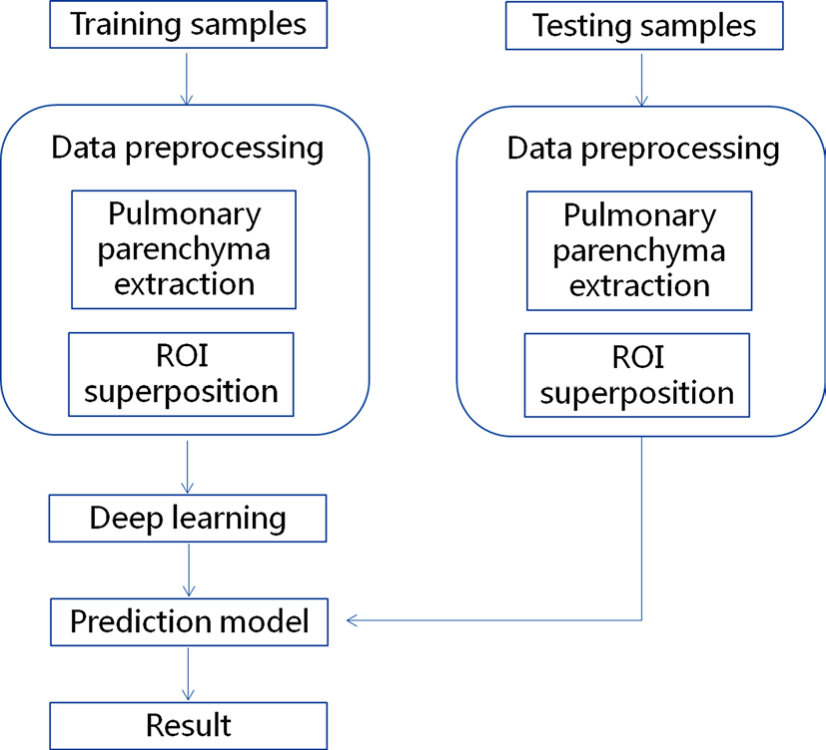
Process of deep learning. *ROI* region of interest

The specific steps involved are shown in Fig. 4.

### Prediction models

AlexNet [14] and GoogLeNet [15] were used to detect pulmonary nodules. The ResNet and the pre-trained ResNet models were used as prediction models for GGOs.

## Analysis

Sensitivity and average false positive rate were used to evaluate the study results. The definitions of some of the terms used in this section are as follows:

- TP (true positive): The number of nodules that were identified as nodules.
- FP (false positive): The number of times that normal tissue was judged as nodules.
- TN (true negative): The number of times that normal tissue was judged as normal tissue.
- FN (false negative): The number of nodules that were judged as normal tissue; this was the number of missed nodules.
- Sensitivity: The proportion of nodules that were correctly classified. The following formula was used: ${\text{Sensitivity}}\, = \,\frac{\text{TP}}{{{\text{TP}}\, + \,{\text{FN}}}}$.
- Average false positive: The average number of nodules missed per patient. The following formula was used: ${\text{Average false positive rate}}\, = \,\frac{\text{FP}}{\text{Patients Numbers}}$.
- Threshold of deep learning testing: After the prediction model analyzed the testing samples, a value of 0 to 1 was the output, which showed the probability of finding pulmonary nodules in the testing. By setting a threshold of deep learning testing, we could distinguish the pulmonary nodules in the testing samples. If the value in the prediction model was higher than that of the threshold, it was believed that the sample contained the pulmonary nodule. If the value was lower, it was considered as normal pulmonary tissue.
- Precision: This indicated that the samples correctly detected accounted for the actual number of GGOs. The formula used was as follows: ${\text{Precision}}\, = \,\frac{\text{TP}}{{{\text{TP}}\, + \,{\text{FP}}}}$.
- True Positive Rate (TPR): This indicated the rate of samples that were correctly identified. The following formula was used: ${\text{True Positive Rate}}\, = \,\frac{\text{TP}}{{{\text{TP}}\, + \,{\text{FN}}}}$.
- F-score: This was used for quantitative analysis of the testing results. The following formula was used: ${\text{F}}\, = \,\frac{{2\, *\,{\text{Precision}}\, *\,{\text{True Positive Rate}}}}{{{\text{Precision}}\, + \,{\text{True Positive Rate}}}}$.

## Results

### Pulmonary nodules

**Fig. 5:**
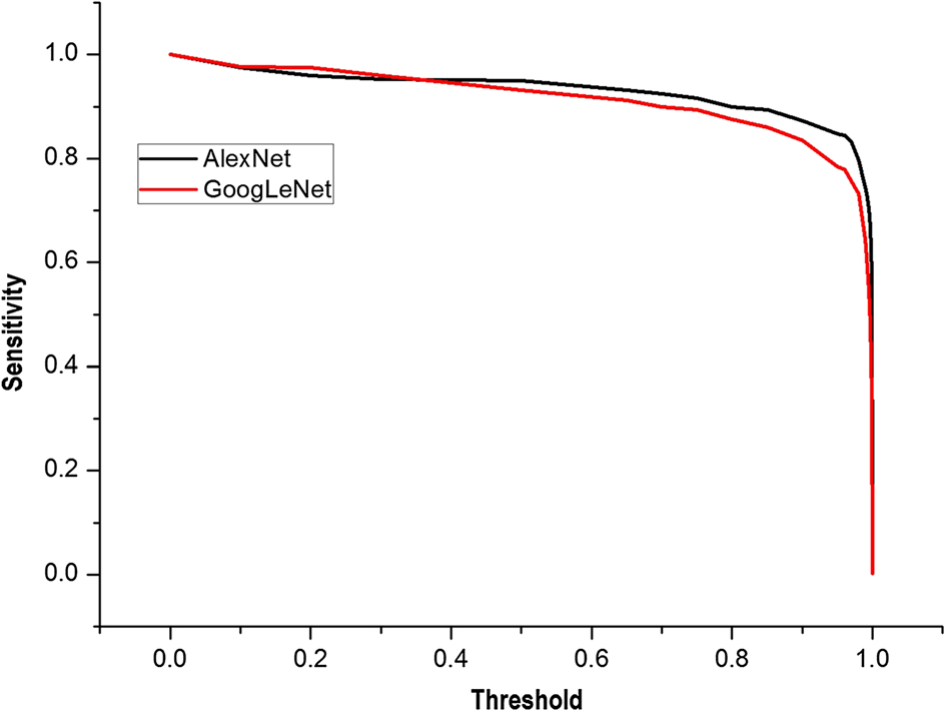
Sensitivity of AlexNet and GoogLeNet with different thresholds. When the threshold was higher than 0.5, the sensitivity of the AlexNet model was slightly better than that of the GoogLeNet model

The models supplied a probability value, which represented the probability that the image was a real nodule. The threshold was used to determine whether a nodule was a real nodule. If the probability value was higher than the set threshold, the image was considered as a real nodule. Thus, by setting different thresholds, the AlexNet and GoogLeNet models had different sensitivities for judging the nodule. The sensitivities of the AlexNet and GoogLeNet models under different thresholds are shown in Fig. 5. It was seen that the sensitivities of AlexNet and GoogLeNet were similar when the threshold was small. When the threshold was higher than 0.5, the sensitivity of the AlexNet model was slightly better than that of the GoogLeNet model.

**Fig. 6:**
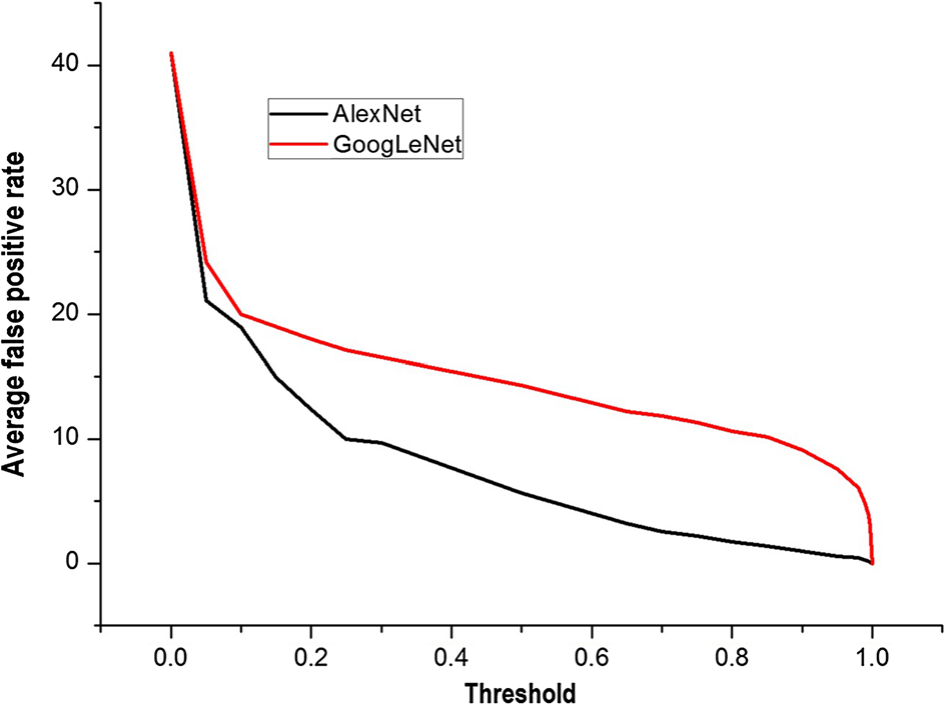
Average false positives with different thresholds of AlexNet and GoogLeNet. The prediction model trained by AlexNet had a better accuracy than that by GoogLeNet

The average FPs of the two models under different thresholds are shown in Fig. 6. It was seen that the average false positive rate of AlexNet was lower than that of GoogLeNet. When the threshold was increased, the average false positive rate of AlexNet decreased faster than that of GoogLeNet. When the threshold was near 0.9, the average FP of GoogLeNet was still about 10. Until the threshold was close to 0.99, the average FP of GoogLeNet was low, but the sensitivity was only about 60–70%. Based on a combination of sensitivity and false positivity, we found that the prediction model trained by AlexNet had better accuracy than that trained by GoogLeNet.

We tested the AlexNet model using a threshold value of 0.5. Results based on the size of pulmonary nodules are shown in Table 1. The results suggest that nodules with diameter less than 10 mm, and especially those smaller than 8 mm, were missed.

**Table 1:** Detection of nodules based on size with AlexNet

Nodule size	TP	FN	Sensitivity (%)
3–5 mm	45	7	86.5
5–8 mm	55	8	87.3
8–10 mm	65	1	98.5
10–20 mm	77	0	100
> 20 mm	63	0	100
Total	305	16	95.0

*FN* false negative, *TP* true positive

### GGOs

**Fig. 7:**
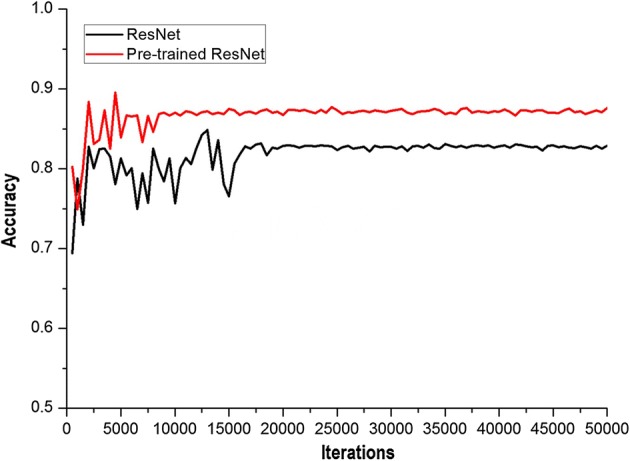
Accuracy with different iteration times of ResNet and pre-trained ResNet. As iterations increased, the accuracies of ResNet and pre-trained ResNet were more stable

During testing of the ResNet and the pre-trained ResNet prediction models to identify GGOs, it was seen that as iterations increased, the accuracies of ResNet and pre-trained ResNet were more stable (Fig. 7). The accuracy of pre-trained ResNet was near 0.87, while that of ResNet was stable near 0.82. By comparing the curves, we determined that pre-trained ResNet not only achieved quick stability, but also had a higher accuracy.

The test results using ResNet and pre-trained ResNet are shown in Table 2. For pre-trained ResNet, when the threshold was 0.6, the maximum value of F-score was 0.87805, TPR was 0.86, precision was 0.897, specificity was 0.903, and false positive rate was 0.097. For ResNet, when the threshold was 0.5, the maximum value of F-score was 0.85528, TPR was 0.897, precision was 0.817, specificity was 0.803, and false positive rate was 0.197. The data showed that the highest F-score obtained by pre-training ResNet was higher than that obtained by ResNet, and almost all the F-scores obtained by pre-training ResNet were better than those when ResNet was used randomly. This shows that when lesser medical image data sets are available, pre-training ResNet could be more effective for improving the training effect of the network and shortening the time required for training to achieve stability. Moreover, it also shows that the accuracy of the prediction model with pre-trained ResNet is better than that with ResNet.

**Table 2:** Classification results of GGOs and non-GGOs by ResNet and pre-trained ResNet

	Threshold	TP	FN	FP	TN	TPR	Precision	F-score
Pre-trained ResNet	0.1	262	31	43	257	0.894198	0.8590164	0.876254
	0.2	255	38	38	262	0.870307	0.8703072	0.870307
	0.3	255	38	33	267	0.870307	0.8854167	0.877797
	0.4	255	38	33	267	0.870307	0.8854167	0.877797
	0.5	253	40	31	269	0.863481	0.8908451	0.87695
	0.6	252	41	29	271	0.86007	0.8968	0.87805
	0.7	250	43	28	272	0.853242	0.8992806	0.875657
	0.8	246	47	25	275	0.83959	0.9077491	0.87234
	0.9	244	49	23	277	0.832765	0.9138577	0.871429
ResNet	0.1	270	23	79	221	0.921502	0.773639	0.841121
	0.2	269	24	72	228	0.918089	0.7888563	0.84858
	0.3	267	26	66	234	0.911263	0.8018018	0.853035
	0.4	265	28	63	237	0.904437	0.8079268	0.853462
	0.5	263	30	59	241	0.89761	0.81677	0.85528
	0.6	260	33	56	244	0.887372	0.8227848	0.853859
	0.7	258	35	53	247	0.880546	0.829582	0.854305
	0.8	256	37	51	249	0.87372	0.8338762	0.853333
	0.9	250	43	46	254	0.853242	0.8445946	0.848896

*FN* false negative, *FP* false positive, *TN* true negative, *TP* true positive, *TPR* true positive rate

## Discussion

In our study, deep learning was combined with CAD to identify pulmonary nodules and GGOs. We have proposed a preprocessing method of RGB superposition in the ROI to effectively increase the differentiation between nodules and normal tissues, and that is the innovation of our research. Our results suggest that the method of deep learning proposed in this study is more sensitive than other CAD systems in recent years, and the average FP is lower than with that with the others (Table 3).

**Table 3:** Comparison of our proposed method with other CADs systems

CAD systems	Sensitivity (%)	Average false positive
Zhang et al. [15]	82.98	11.76
Ye et al. [16]	90.2	8.20
Choi et al. [17]	95.28	2.27
Setio et al. [18]	90.1	4.00
Ma et al. [19]	88.9	4.00
Liu et al. [20]	89.4	2.00
Our study	95.0	5.62

*CAD* computer-aided diagnosis

The detection rate of GGOs is increasing because of the widespread use of multislice spiral CT and CT screening for lung cancer detection [18–23]. Pathologically, ground-glass nodules can be either benign or malignant lesions. Several studies have shown that persistent GGOs have a high risk of malignancy. Compared with solid nodules, the type of malignancy in GGOs (nonsolid nodules) is predominantly adenocarcinoma or precancerous lesion [24, 25]. Therefore, early identification of pulmonary nodules, especially GGOs, has a great diagnostic and therapeutic significance in patient management.

Deep learning and CAD have been the new research hotspots in recent years. AlexNet is a classical open source convolutional neural network algorithm. It was a winner of the ImageNet large scale visual recognition challenge (ILSVRC) in 2012 [14]. The Network structure of GoogLeNet is more complex, and it was the winner of the ILSVRC in 2014 [15]. ResNet is proposed by the Microsoft Research Institute; it can effectively solve the problem of accuracy getting saturated with increasing depth [16]. These are the reasons why we chose these specific CAD systems for our study.

With regard to the utility of deep learning for the detection and classification of pulmonary nodules and GGOs, we did find that the method of deep learning proposed in our study is feasible; however, there are still some shortcomings and improvements will need to be made. First, compared with other CAD system experiments, the FPs in our study are a little high; we intend to try to reduce it by introducing the pre-trained deep neural network algorithm weights in ImageNet data to initialize. Second, deep learning requires training data with large sample sizes. The samples of pulmonary ground-glass opacity used in our study are still not adequate. We will continue the experiment with regard to GGOs, with a higher sample size, and we believe that the accuracy of deep learning could improve further. We also plan to collect the pathological results of GGOs and use them in deep learning. We hope that in addition to identifying pulmonary nodules correctly, deep learning can provide a preliminary diagnosis of nodules as benign or malignant in order to make the results of CAD more applicable in clinical practice.

## Conclusion

The method of deep learning proposed in this study is more sensitive and has a lower average FP compared to other systems, which would effectively increase the differentiation between nodules and normal tissues, and help early identification of pulmonary nodules, especially GGOs.

## Abbreviations

CAD: computer-aided diagnosis
CAFFE: convolutional architecture for fast feature embedding
CT: computed tomography
GGO: ground-glass opacity
FN: false negative
FP: false positive
ILSVRC: ImageNet large scale visual recognition challenge
LIDC-IDRI: Lung Image Database Consortium and Image Database Resource Initiative
RGB: red, green, and blue channels
ROI: region of interest
TN: true negative
TP: true positive
TPR: true positive rate
